# The Thirtieth Annual Commencement Exercises of Rush Med. College

**Published:** 1873-03

**Authors:** 


### *The Thirtieth Annual Commencement Exercises of Rush Med. College*

Were held in Central Hall, corner Wabash Av. and 22nd St., on Wednesday evening, Feb. 19, 1873. A crowded audience room bore witness to the interest manifested by physicians and the friends of the College. The Rt. Rev. Bishop Foley opened the exercises with prayer, after which Professor Freer, President of the College, conferred the diplomas on the following gentlemen, who had complied with the rules and regulations of the College for the secural of the degree of Doctor in Medicine:

| | | |
|---|---|---|
| 1. J. I. Ashbaugh. | 12. C. M. Dodge. | 23. F. A. Hess. |
| 2. Franklin Bedford. | 13. W. L. Duffin. | 24. C. F. King. |
| 3. W. H. Battin. | 14. E. M. Enfield. | 25. E. A. Kittell. |
| 4. J. M. Barclay. | 15. D. W. Edmiston. | 26. J. A. Kettring. |
| 5. H. C. Bostwick. | 16. J. W. Evans. | 27. M. H. Luken. |
| 6. J. B. Browning. | 17. John Grass. | 28. F. E. Lewis. |
| 7. C. C. Birney. | 18. C. V. Von Hiddessen. | 29. N. A. Loofbourow. |
| 8. J. H. Cristler. | 19. W. A. Horton. | 30. G. B. Little. |
| 9. C. H. Carey. | 20. C. H. Hamilton. | 31. C. L. Myers. |
| 10. E. B. Crommett. | 21. W. J. Hart. | 32. P. M. Mendenhall. |
| 11. F. B. Corbett. | 22. A. J. Hynds. | 33. Geo. McCulloch. |

34. M. G. McLean.
35. J. H. Mear.
36. O. C. Oliver.
37. D. W. Pearson.
38. W. W. Rurk.
39. G. W. Reynolds.
40. H. R. Riddle.
41. M. G. Sloan.
42. F. E. Sherman.
43. F. Shimonek.
44. J. F. Shaefer.
45. C. H. Smith.
46. J. J. Stone.
47. G. D. Swaine.
48. E. R. Smith.
49. J. N. Starr.
50. D. M. Slemmons.
51. K. T. Stabeck.
52. M. Shoemaker.
53. J. S. Thompson.
54. R. N. Turner.
55. H. J. Thomas.
56. W. W. Wood.
57. J. G. Walker.
58. E. B. Weston.
59. G. C. Wellner.
60. H. A. Winter.
61. J. T. Walker.

POST-MORTEM DEGREE—Sanford O. Alford.

HONORARY DEGREE—A. Reeves Jackson, M.D., and Philip Adolphus, M.D., of Chicago; Chas. L. Allen, M.D., of Rutland, Vt., and Thomas O. Catlen, M.D., of New York City.

In accordance with a long established custom, the President then delivered his annual address to the class. He commended them for their high scholarship, excellent deportment, and uniform courtesy to their teachers, congratulating them on having so successfully and creditably passed their final examinations. Their attention was seriously called to the fact that they must *study*, all their lives, keeping up with the latest developments of the profession, must buy books and instruments so far as their means allow, not forgetting to own, soon as possible, their microscope and become adepts in its use.

At the conclusion of the President's address, Dr. E. R. Smith, on behalf of the class, delivered the class valedictory, thanking the Faculty for what they had done for them, and promising unswerving fidelity to their Alma Mater.

Adjunct Prof. Walter Hay, M.D., was then introduced, and proceeded to deliver an unusually pleasing address, replete with the advice of a mature experience, elegantly expressed. Our space admits only of quoting one of the most exquisite perorations it has been our good fortune to listen to:

"To-day terminates the period of your pupilage, but not of your student life; that is just begun, and you have been looking forward to the commencement day as to the beginning of a new era in your lives, in which you would be no longer medical students. Many years ago I used to stand at the gateway of a noble old mansion in a far distant State, beneath the shade of a grand old aspen tree upon whose glistening bark the scholarly owner had carved in bold Roman characters the single word 'Vale'; and when I passed through that gateway out upon the dusty highway beyond, the quivering leaves of the aspen overhead seemed to whisper 'Vale.' To-night I seem to stand, like that old tree, at the threshold of your Alma Mater, to say to you in the name of the faculty of Rush Medical College, 'Vale,' Farewell. On the other side of that tree was another word which greeted the eye of the guest about to enter those hospitable shades, and that word I repeat to you in the name of the medical profession, whose doors are now open to you, 'Salve,' Welcome."

A large, beautiful desk was then presented to Prof. Allen by the class, Graduate Frank E. Lewis, M.D., making the presentation speech. Dr. Allen received the keys with one of his happy speeches, showing unmistakable signs of not having had any time to prepare a little extemporaneous (!) speech for the occasion.

After the benediction by Bishop Foley, the audience dispersed.

J. H. ETHERIDGE, *Asst. Sec. R. M. C.*

